# Dissolution and Early Hydration Interaction of C_3_A-C_4_AF Polyphase in Water and Aqueous Sulfate Solutions

**DOI:** 10.3390/ma18143399

**Published:** 2025-07-20

**Authors:** Shaoxiong Ye, Pan Feng

**Affiliations:** 1 College of Civil Engineering, Huaqiao University, Xiamen 361021, China; 2 Jiangsu Key Laboratory of Construction Materials, School of Materials Science and Engineering, Southeast University, Nanjing 211189, China; 3 State Key Laboratory of High Performance Civil Engineering Materials, Nanjing 210008, China

**Keywords:** C_3_A, C_4_AF, co-dissolution, hydration, kinetics

## Abstract

The concurrent dissolution and early hydration of tricalcium aluminate (C_3_A) and tetracalcium aluminoferrite (C_4_AF) critically govern early-stage reaction dynamics in Portland cement systems. However, their mutual kinetic interactions during reaction, particularly sulfate-dependent modulation mechanisms, remain poorly understood. Using in-situ digital holographic microscopy (DHM), this study resolved their interaction mechanisms during co-dissolution in aqueous and sulfate-bearing environments. Results reveal asymmetric modulation: while C_4_AF’s dissolution exhibited limited sensitivity to C_3_A’s presence, C_3_A’s kinetics were profoundly altered by C_4_AF through sulfate-concentration-dependent pathways, which originated from two competing C_4_AF-mediated mechanisms: (1) suppression via common-ion effects, and (2) acceleration through competitive sulfate species adsorption. These mechanistic insights would provide a roadmap for optimizing cementitious materials through optimized reaction pathways.

## 1. Introduction

In Portland cement systems, intermediate clinker phases tricalcium aluminate (C_3_A) and tetracalcium aluminoferrite (C_4_AF) critically govern early-age hydration kinetics and properties [1,2,3]. While enabling energy-efficient clinker production [4,5], they introduce fundamental reactivity trade-offs: C_3_A’s rapid hydration risks flash setting while C_4_AF variability complicates sulfate balancing [6,7]. However, their co-reaction dynamics remain inadequately resolved due to the multiphase nature of Portland cement systems, where concurrent dissolution of multiple components alongside precipitation of hydration products obscures mechanistic analysis [8,9]. Resolving the interdependent kinetics of C_3_A and C_4_AF is thus imperative for controlling cement’s setting behavior and enabling concrete optimization through optimized reaction pathways.

The inherent complexity primarily stems from the dynamic concurrent dissolution and hydration of intermediate phases upon water contact [10]. Within seconds of mixing, simultaneous release of Ca^2+^, Al^3+^, and Fe^3+^ ions establishes competitive ion-exchange environments where reaction pathways diverge according to local concentration gradients [11,12,13]. To overcome this complexity, isolating C_3_A and C_4_AF enables precise tracking of individual reaction processes, providing deeper mechanistic insights into actual cement systems [14,15,16,17,18,19]. Consequently, research utilizing pure intermediate phases constitutes a fundamental methodology in cement chemistry. Fundamentally, both C_3_A and C_4_AF follow analogous sulfate-dependent hydration sequences dominated by sequential ettringite-monosulfate (AFt-AFm) formation [17,18]. Specifically, in pure water, both phases undergo rapid dissolution followed by violent hydration, forming metastable hydrates (CAH_10_/C_2_AH_8_ for C_3_A; analogous Fe-substituted phases for C_4_AF) that eventually convert to thermodynamically stable phase hydrogarnet, causing flash set through instantaneous precipitation of interlocking crystals [20,21]. Some molecular simulations have revealed that intermediate phases’ surfaces promote water dissociation, facilitating calcium ion desorption through sequential formation of complexes that reprecipitate on Al-enriched layers via interface-coupled dissolution–reprecipitation [22,23,24]. With gypsum, gypsum initially directs ettringite formation with both phases; subsequent sulfate depletion drives conversion to monosulfate [14,15]. Crucially, this sulfate-mediated regulation governs setting kinetics and strength development, rendering gypsum indispensable for achieving optimal workability in Portland cement. This regulation is usually attributed to adsorption of ions or complexes onto the hydrating surface, as evidenced by macro/microscopic studies [25,26]. Bulk solution analyses also support this mechanism, indicating that Al-rich leached layer formation and subsequent ion pair adsorption mediate dissolution inhibition [27,28].

Comparative studies confirm C_4_AF exhibits inherently slower hydration kinetics than C_3_A due to iron substitution-mediated retardation [20,29]. The high early reaction rate is primarily attributed to rapid dissolution, with recent work of Ye et al. [30] firstly quantitatively characterizing C_3_A’s dissolution kinetics in pure water and determining its dissolution rate ranging in 0.5~2 mmol·m^−2^·s^−1^ (several orders of magnitude greater than that of C_4_AF [31]). For the intermediate phases, previous studies have demonstrated that sulfates exert ion-specific retardation effects on their dissolution: among Na_2_SO_4_, MgSO_4_, and CaSO_4_, Na_2_SO_4_ has the least while CaSO_4_ has the strongest retardation effect [31,32]. Furthermore, dissolution rates and mechanisms depend on solution undersaturation: in water, dissolution proceeds rapidly via etch pit formation and vacancy islands, whereas in saturated solutions, reduced driving force leads to step retreat from pre-existing etch pits [33,34]. Nevertheless, despite these recent studies generating fundamental knowledge on the hydration reactions, cement hydration is not simply the sum of individual phase reactions, but the result of interactions between all phases [35,36,37,38]. The co-dissolution behavior of aluminate phases governs early cement hydration kinetics yet defies prediction from isolated phase studies due to multiphase interactions. Competitive sulfate consumption, which prioritizes C_3_A reactivity over C_4_AF, was evidenced by near-complete ettringite formation from C_3_A and residual unreacted C_4_AF due to iron exclusion from AFt phases [39]. This sulfate competition is further modulated by supplementary cementitious materials (SCMs), as aluminum from SCMs increases sulfate demand, accelerating depletion and altering C_3_A/C_4_AF hydration dynamics. Notably, pozzolanic additives exhibit divergent effects by accelerating C_3_A–gypsum reactions in water while retarding them in Ca(OH)_2_-saturated solutions [38]. Meanwhile, ye’elimite interactions reveal crystal-structure-dependent inhibition: orthorhombic ye’elimite with C_4_AF/gypsum yields ettringite and AFm mixtures, while pseudocubic solid solutions strongly retard hydration, promoting dominant ettringite formation [15]. These phase interactions all complicate the co-dissolution behavior of C_3_A/C_4_AF, resulting in an inadequately resolved underlying mechanism. Therefore, characterizing C_3_A-C_4_AF polyphase systems’ dissolution behaviors still remains challenging due to their remarkable reactivities and unquantified interaction mechanisms.

Building upon prior single-phase investigations of C_3_A’s and C_4_AF’s dissolution and early hydration kinetics via digital holographic microscope (DHM) [30,31,32], this study extends this approach to resolve the unresolved multiphase coupling effects in C_3_A-C_4_AF polyphase systems under controlled sulfate conditions. By correlating real-time dissolution topographies and dissolution rates in aqueous and sulfate environments, we establish a mechanistic framework to quantify sulfate-dependent competitive pathways governing polyphase systems’ co-hydration, aiming to decouple the interactions during co-reaction and quantify the sulfate-modulated reaction kinetics.

## 2. Materials and Methods

C_3_A powder (median particle size D_50_ = 9.083 μm, purity 96.73%) and C_4_AF powder (D_50_ = 11.353 μm, purity 99.65%) were prepared by solid-state reaction in accordance with [40]. These powders were blended in a 7:3 mass ratio for 2 h using a Turbula T2F mixer (Willy A. Bachofen AG, Muttenz, Switzerland), then pressed into compacts. Polished C_3_A-C_4_AF polyphase pellets were fabricated by hot pressing these compacts in a spark plasma furnace (HP-D5, FCT, Frankenblick, Germany) and used as the samples for the flow-through dissolution experiments. The sintering process involved heating to 1150 °C (to avoid severe melting [40]) at 100 °C·min^−1^ while pressure concurrently increased from 15 MPa to 40 MPa. The pellets were held at this maximum temperature and pressure for 15 min, followed by meticulous polishing.

For the investigation of the co-dissolution of C_3_A-C_4_AF in sulfate environments, sulfate aqueous solutions of Na_2_SO_4_, MgSO_4_, and CaSO_4_ were prepared by dissolving appropriate amounts of sulfate salts in ultrapure water (resistivity > 18 MΩ·cm at 25 °C) to be used as the testing solutions. To enable direct comparison of reported dissolution rates for individual C_3_A or C_4_AF in sulfate solutions [31,32], Na_2_SO_4_ and MgSO_4_ solutions were prepared at concentrations of 1 mmol·L^−1^ and 30 mmol·L^−1^. Due to gypsum’s poor solubility, CaSO_4_ solutions were prepared at lower concentrations of 1 mmol·L^−1^ and 10 mmol·L^−1^.

Prior to flow-through dissolution experiments, polished C_3_A-C_4_AF polyphase pellets were partially coated with a platinum reference layer (~20 nm thickness) using a sputter coater (Q150T, Quorum Technologies, Laughton, UK). A R-2203 digital holographic microscope (Lyncée Tec, Lausanne, Switzerland) in reflection mode was employed to track dissolution behaviors in various solutions, following established protocols [30,31,32]. Experiments were conducted at 20 ± 1 °C with a constant flow rate of 34 mL·min^−1^ to prevent hydrate precipitation during testing. During dissolution, the surface heights of C_3_A and C_4_AF undergo varying degrees of change. By analyzing topography shifts, comparing height differences (∆*h*, in units of m) between exposed and masked areas over time intervals (∆*t*, in units of s), and using their molar volumes and height evolution rates, the individual dissolution rates can be calculated as follows [31],

(1) $$R=\frac{\Delta h}{\Delta t}\frac{1}{V_{m}}$$

where *R* is the dissolution rate of C_3_A or C_4_AF (in units of mol·m^−2^·s^−1^), ∆*h* is the surface height change within ∆*t*, and *V*_m_ is the molar volume of C_3_A (8.91 × 10^−5^ m^3^·mol^−1^) or C_4_AF (1.28 × 10^−4^ m^3^·mol^−1^) [41].

After the specified reaction durations, some dissolved C_3_A-C_4_AF polyphase pellets were collected for morphological analysis. Upon removal from the reaction cell, they were promptly dried using compressed air. An FEI 3D scanning electron microscope (FEI, Hillsboro, OR, USA), equipped with an EDAX system (AMETEK, Berwyn, PA, USA) and operating in secondary electron mode at an accelerating voltage of 20 kV, was employed to examine the morphological alterations of C_3_A and C_4_AF during the reaction. Before testing, a ~50 nm thick platinum layer was sputter-coated on the pellet surfaces.

## 3. Results and Discussion

### 3.1. Dissolution Interaction in Water

**Figure 1:**
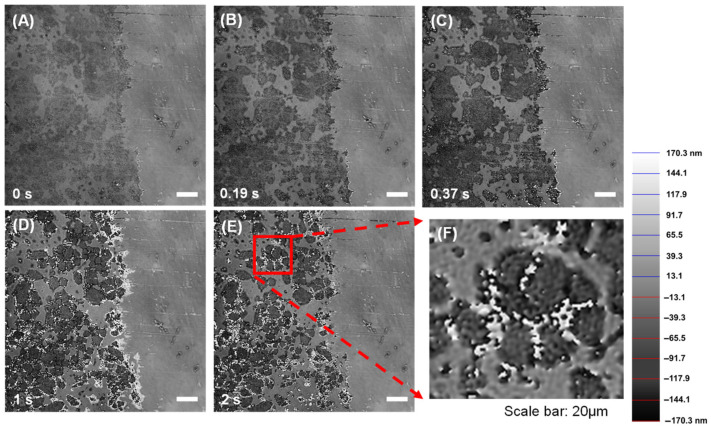
Phase images of C_3_A-C_4_AF pellet acquired by DHM when dissolving in ultrapure water with a flow rate of 34.0 mL·min^−1^ for (**A**) 0 s, (**B**) 0.19 s, (**C**) 0.37 s, (**D**) 1 s, and (**E**,**F**) 2 s.

Figure 1 presents phase diagrams of a C_3_A-C_4_AF polyphase pellet after dissolution for varying durations in flowing water (34.0 mL·min^−1^), derived from height-inclusive DHM images. These diagrams delineated the exposed area (left) from the inert platinum-coated area (right). Within the exposed region, distinct dissolution behaviors were observed: darker areas exhibited rapid height changes, indicative of faster dissolution, whereas brighter areas remained relatively stable in height, suggesting slower dissolution. This visual contrast directly implies a significant difference in dissolution rates between the two regions. Given the well-established, substantial difference in dissolution kinetics between C_3_A and C_4_AF [30,31], it is reasonable to attribute the rapidly dissolving darker areas to C_3_A and the more stable brighter areas to C_4_AF. This phase assignment is further supported by Figure 1F, which highlights that the most rapid dissolution of C_3_A occurred specifically at its grain boundaries [42]. Additionally, the grain size of individual C_3_A particles within the co-sintered pellet was measured to be approximately 20 μm. This value aligns closely with the grain size observed in our previously sintered pure C_3_A pellets [30]. Therefore, this consistency in grain size indicates that the co-sintering process with C_4_AF did not significantly alter the crystal structure of the components.

**Figure 2:**
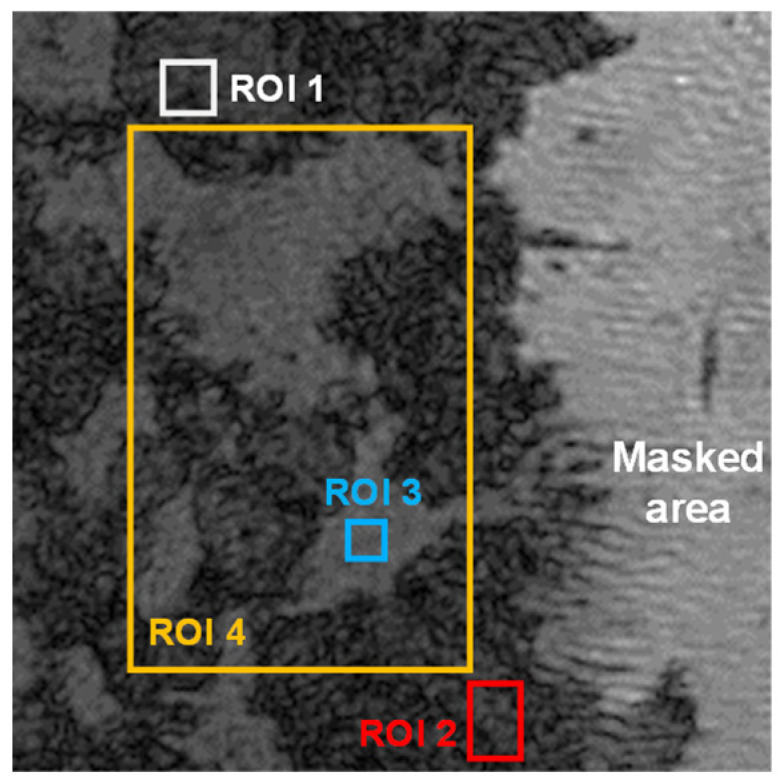
The selection of ROI 1, ROI 2, ROI 3, and ROI 4 in an intensity image of C_3_A-C_4_AF pellet acquired by DHM with a size of 70 μm × 70 μm.

**Figure 3:**
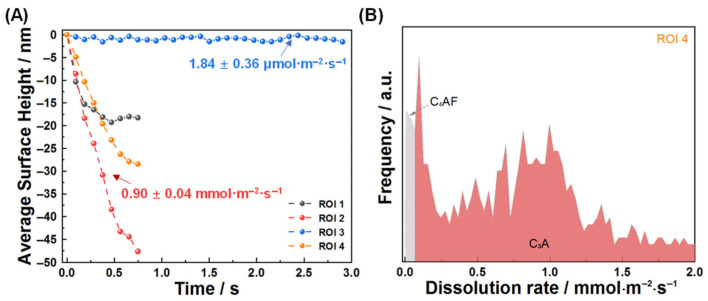
When dissolving in ultrapure water, (**A**) average surface height changes of ROI 1, ROI 2, ROI 3, and ROI 4, and (**B**) dissolution rate distribution spectra of ROI 4 within the first 0.2 s dissolution.

The distinct optical reflectivity of C_3_A and C_4_AF [43] allows for direct phase segmentation within DHM light intensity images (Figure 2), enabling independent characterization of each phase’s co-dissolution behavior. To quantify this behavior, we analyzed four regions of interest (ROI 1–4) within a representative 70 μm × 70 μm area, tracking their surface height evolution and dissolution rate spectra (Figure 3). Analysis of surface height evolution (Figure 3A) revealed a significantly lower rate in ROI 3 (identified as C_4_AF) compared to the other regions. This observation directly demonstrates the markedly different dissolution kinetics between C_3_A and C_4_AF. Further corroborating this difference, the dissolution rate frequency spectrum for ROI 4 (Figure 3B) clearly distinguished the high-dissolution-rate zone dominated by C_3_A from the low-dissolution-rate zone dominated by C_4_AF. Quantitatively, within the C_3_A-C_4_AF polyphase pellet, the dissolution rate of C_4_AF was measured at 1.84 μmol·m^−2^·s^−1^, nearly equivalent to the dissolution rate reported previously for pure C_4_AF [31], while C_3_A (in ROI 2) dissolved at a rate of 0.90 mmol·m^−2^·s^−1^. It is important to note, however, a methodological consideration affecting the presented dissolution rates: For data processing convenience, the dissolution rates of all pixels within ROI 4 were calculated using the molar volume of C_3_A. Due to the inherent difference in molar volumes between C_3_A and C_4_AF [41], this approach resulted in a slight overestimation of the dissolution rates specifically in the low-dissolution-rate areas (C_4_AF) depicted in Figure 3.

Given that the sintering protocol for the C_3_A-C_4_AF polyphase pellet was identical to that used for the pure C_4_AF pellet [31], and considering that the measured dissolution rate of C_4_AF within the pellet (Figure 3) showed no significant difference from that of the pure C_4_AF pellet [31], this study primarily focused on comparing the dissolution rate of C_3_A within the polyphase pellet to that of the pure C_3_A pellet. Although the lower sintering temperature of the polyphase pellet (vs. pure C_3_A [30,32]) might predict increased C_3_A defect density and reactivity, the near-identical grain size of C_3_A in both systems (Figure 1 in this study and Figure 1 in [30]) suggested minimal differences in dissolution kinetics.

**Figure 4:**
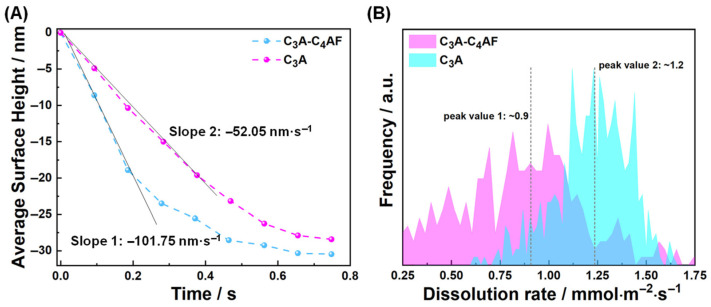
Comparison of (**A**) the average surface height changes and (**B**) the dissolution rate distribution spectra within the first 0.2 s dissolution between RIO 4 and pure C_3_A when dissolving in water.

Figure 4 presents a comparative analysis of surface height evolution and dissolution rate frequency spectra between ROI 4 (within the polyphase pellet) and the pure C_3_A pellet [30] during dissolution. Crucially, while the slower mean height reduction in ROI 4 (Figure 4A) stems from methodological averaging over C_4_AF regions, Figure 4B reveals a material-specific trend: the high-rate dissolution peak (C_3_A-dominated) shifts distinctly leftward in the composite—indicating suppressed dissolution kinetics versus pure C_3_A. This spectral shift provides direct evidence of slight dissolution inhibition of C_3_A within the polyphase matrix. This inhibition is attributed to C_3_A’s sensitivity to solution ion concentrations. Ions released from co-dissolving C_4_AF likely induce a common-ion effect that suppresses C_3_A’s dissolution, a phenomenon frequently observed in mineral dissolution studies [44,45].

### 3.2. Dissolution and Early Hydration Interaction in Sulfate Aqueous Solutions

**Figure 5:**
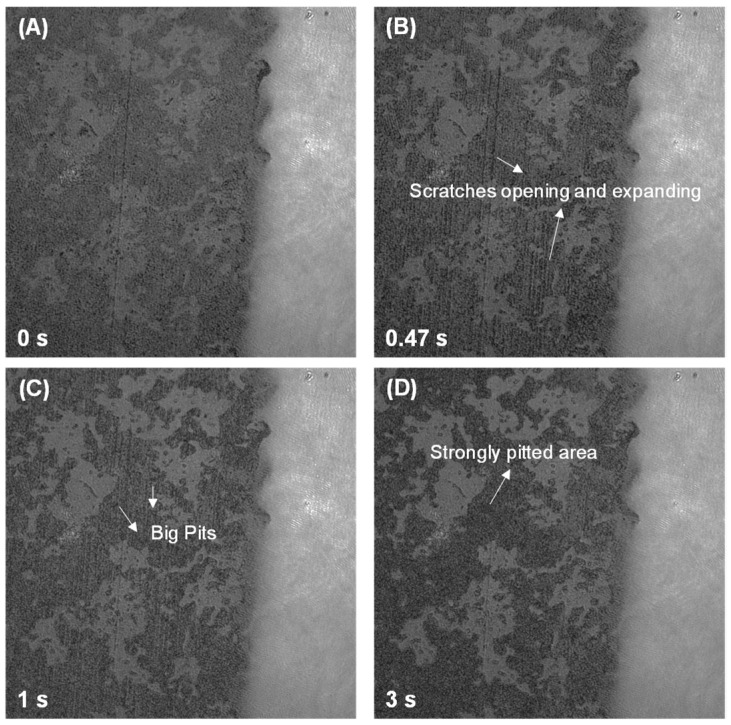
Intensity images of a C_3_A-C_4_AF polyphase pellet acquired by DHM with a size of 192.4 μm × 192.4 μm when dissolving in a 1 mmol·L^−1^ CaSO_4_ solution for (**A**) 0 s, (**B**) 0.47 s, (**C**) 1 s, and (**D**) 3 s.

**Figure 6:**
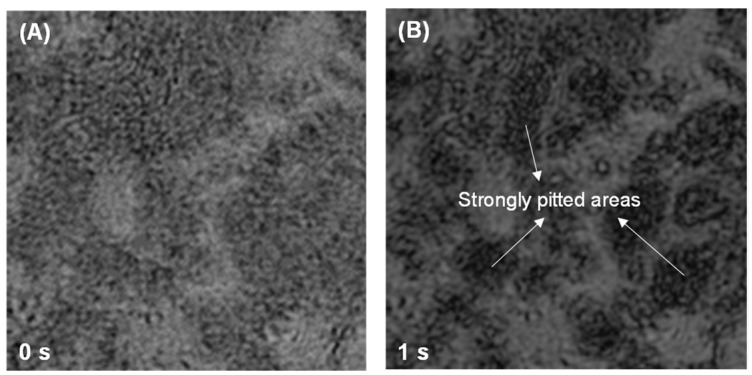
Intensity images of a C_3_A-C_4_AF polyphase pellet acquired by DHM with a size of 70 μm × 70 μm when dissolving in a 10 mmol·L^−1^ CaSO_4_ solution for (**A**) 0 s and (**B**) 1 s.

**Figure 7:**
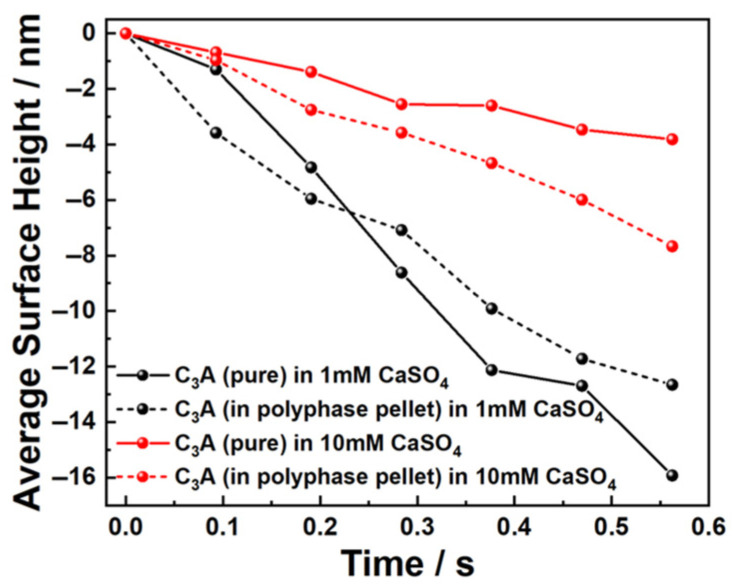
Average surface height changes of pure C_3_A and C_3_A in a C_3_A-C_4_AF polyphase pellet when dissolving in CaSO_4_ solutions.

This study also employed DHM to investigate the sulfate-modified dissolution behavior in C_3_A-C_4_AF polyphase pellets, with focus on C_3_A’s kinetics due to C_4_AF’s markedly lower dissolution rate. Figure 5 illustrates the morphological evolution of the polyphase pellet in 1 mmol·L^−1^ CaSO_4_ solution. After 3 s of dissolution, C_4_AF exhibited negligible morphological changes, while C_3_A underwent rapid transformation manifested by propagating scratches and the formation/growth of etch pits, resulting in significant surface roughening. Similarly, in 10 mmol·L^−1^ CaSO_4_, C_3_A developed pronounced corrosion features and surface roughening within 1 s, as shown in Figure 6. Critical analysis of surface height evolution (Figure 7) demonstrates sulfate-dependent rate inversion: in 1 mmol·L^−1^ CaSO_4_, C_3_A’s dissolution was slower within the polyphase pellet than in pure C_3_A, whereas in 10 mmol·L^−1^ CaSO_4_, C_3_A’s dissolution was faster within the polyphase pellet than in pure C_3_A.

This concentration-dependent divergence was supposed to originate from two competing interactions during co-dissolution: (1) a suppressive effect whereby ions released from dissolving C_4_AF induce a common-ion effect inhibiting C_3_A’s dissolution, and (2) an accelerative effect arising from C_4_AF’s positively charged surface competing with C_3_A for sulfate ions (SO_4_^2−^) and ion pair (e.g., CaSO_4_^0^) adsorption, thereby attenuating sulfate-mediated inhibition of C_3_A’s dissolution. Cross-validation with pure C_3_A’s dissolution kinetics [32] and Figure 4 confirm that sulfate inhibition dominated at high concentrations, substantially exceeding the suppressive influence of C_4_AF-derived ions. Consequently, under dilute sulfate (1 mmol·L^−1^), the common-ion effect derived from C_4_AF, reduced C_3_A’s dissolution; conversely, in concentrated sulfate (10 mmol·L^−1^), C_4_AF’s competitive adsorption mitigated intrinsic sulfate inhibition, accelerating C_3_A’s dissolution in the polyphase pellet.

**Figure 8:**
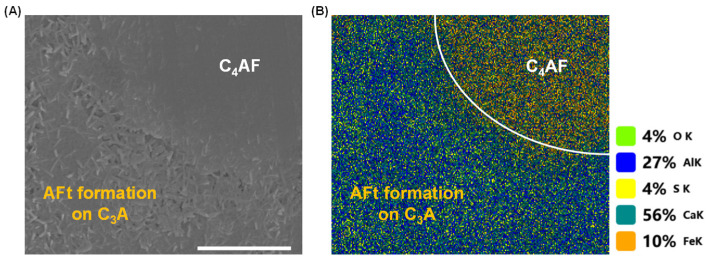
(**A**) Morphology and (**B**) element mapping of a C_3_A-C_4_AF polyphase pellet after reacting in a 10 mmol·L^−1^ CaSO_4_ solution for 2 s. Scale bar, 5 μm.

**Figure 9:**
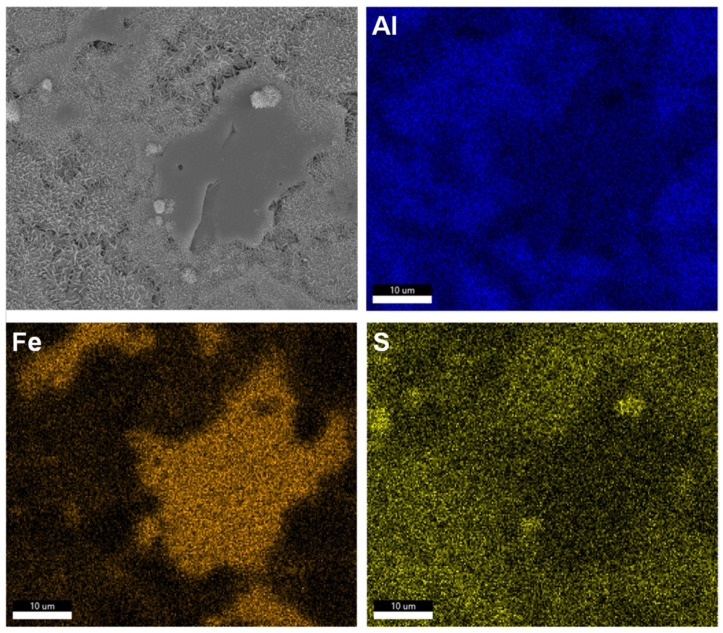
Morphology and element mappings of a C_3_A-C_4_AF polyphase pellet after reacting in a 10 mmol·L^−1^ CaSO_4_ solution for 1 min. Scale bar, 20 μm.

Although both C_3_A and C_4_AF can adsorb sulfate species [28,46], the faster dissolution kinetics of C_3_A lead to accelerated accumulation of dissolved ions at its interface. Consequently, when the polyphase pellet hydrated in calcium sulfate solution, needle-like AFt crystals [47] preferentially nucleated and grew on C_3_A’s surface, as demonstrated in Figure 8. As dissolution progressed, AFt deposition progressively covered C_3_A’s surface while gradually developing on C_4_AF’s surface, as shown in Figure 9.

**Figure 10:**
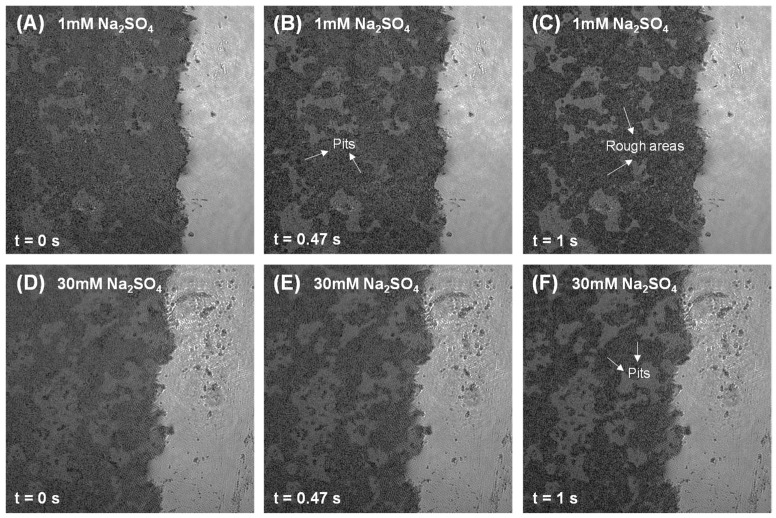
Intensity images of a C_3_A-C_4_AF polyphase pellet acquired by DHM with a size of 192.4 μm × 192.4 μm when dissolving in a 1 mmol·L^−1^ Na_2_SO_4_ solution for (**A**) 0 s, (**B**) 0.47 s, and (**C**) 1 s, and in a 30 mmol·L^−1^ Na_2_SO_4_ solution for (**D**) 0 s, (**E**) 0.47 s, and (**F**) 1 s.

**Figure 11:**
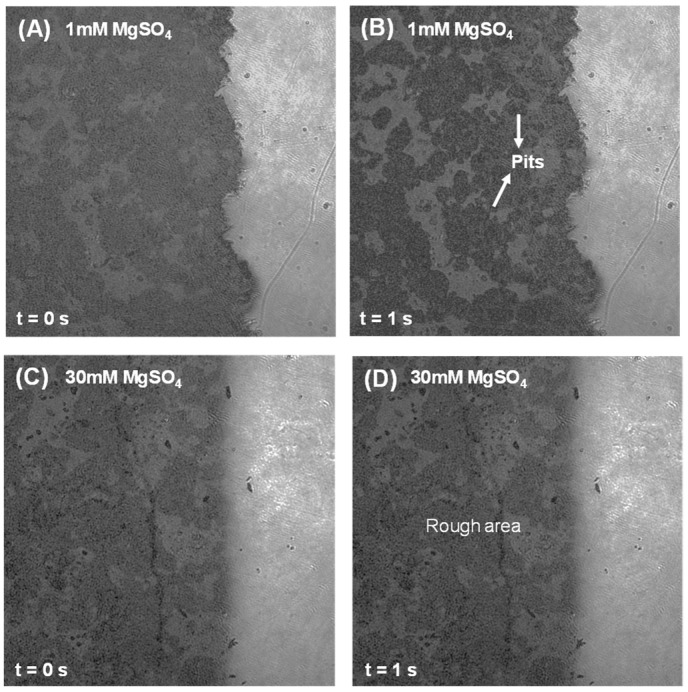
Intensity images of a C_3_A-C_4_AF polyphase pellet acquired by DHM with a size of 192.4 μm × 192.4 μm when dissolving in a 1 mmol·L^−1^ MgSO_4_ solution for (**A**) 0 s and (**B**) 1 s, and in a 30 mmol·L^−1^ MgSO_4_ solution for (**C**) 0 s and (**D**) 1 s.

Furthermore, this study also investigated the influence of sulfate-counter cations (Na^+^, Mg^2+^) on C_3_A’s dissolution in the C_3_A-C_4_AF polyphase pellets. As demonstrated in Figure 10 and Figure 11, in both sodium sulfate (Na_2_SO_4_) and magnesium sulfate (MgSO_4_) solutions at low concentrations, C_3_A within the polyphase underwent rapid surface transformation characterized by abundant etch pit formation and progressive roughening. Conversely, minimal surface alteration occurred under high-concentration sulfate conditions, consistent with prior observations [32].

**Figure 12:**
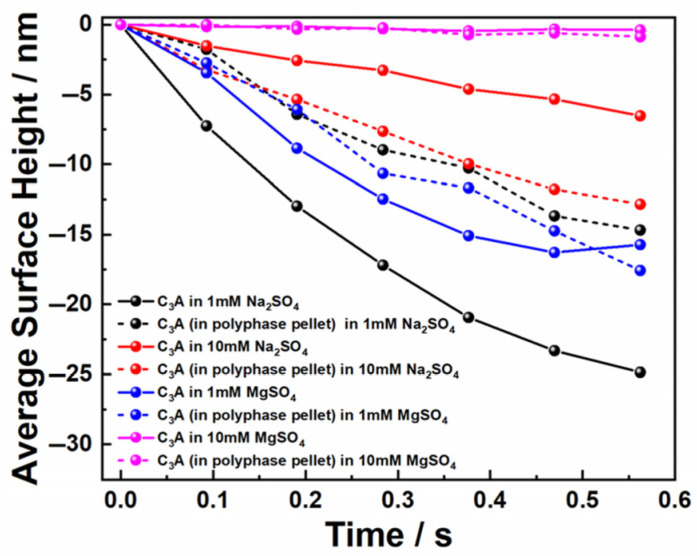
Average surface height changes of pure C_3_A and C_3_A in a C_3_A-C_4_AF pellet when dissolving in Na_2_SO_4_ and MgSO_4_ solutions.

Similarly, the dissolution differences between pure C_3_A and C_3_A within the polyphase pellet were compared in both sulfate solutions, with quantitative results presented in Figure 12. A consistent pattern emerged: specifically, in dilute sulfate solutions, C_3_A’s dissolution slowed within the polyphase system, whereas in concentrated sulfate solutions, dissolution accelerated—mirroring the behavior observed in calcium sulfate solutions. This concentration-dependent inversion was governed by the competing interaction mechanisms of C_4_AF, as established previously.

**Figure 13:**
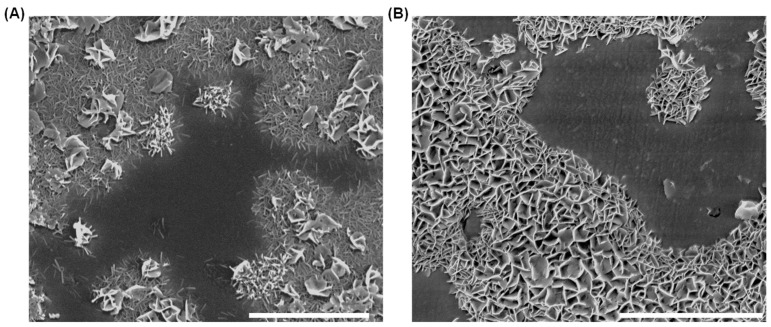
Morphologies of a C_3_A-C_4_AF polyphase pellet after reacting in a 30 mmol·L^−1^ Na_2_SO_4_ solution for (**A**) 2 s and (**B**) 1 min. Scale bar, 10 μm.

Although C_3_A exhibited identical dissolution rate patterns in these sulfate solutions as observed in calcium sulfate solutions, the composition of surface products differed significantly. As shown in Figure 13, flaky AFm phases [47] rapidly formed and progressively became the dominant product in both sulfate solutions, which contrasts with the product assemblages formed in calcium sulfate solutions.

## 4. Conclusions

This study elucidates the dissolution and early hydration interactions between C_3_A and C_4_AF, revealing sulfate-dependent asymmetric modulation: C_4_AF’s dissolution exhibited minimal sensitivity to C_3_A, whereas C_3_A’s reaction kinetics were profoundly altered by C_4_AF via sulfate-concentration-dependent pathways. A critical sulfate-concentration threshold governed reaction pathways: C_3_A’s dissolution slowed in the polyphase versus pure phase at 1 mM SO_4_^2−^, yet accelerated at 10 mM SO_4_^2−^. These phenomena originate from two competing mechanisms derived from C_4_AF: (1) suppression through common-ion effects, and (2) acceleration via competitive adsorption of sulfate species. By quantifying the co-reaction dynamics of C_3_A-C_4_AF in water/sulfate solutions, this study contributes to improved hydration modeling accuracy and advances mechanistic understanding of cement reactivity.

## Data Availability

The original contributions presented in this study are included in the article. Further inquiries can be directed to the corresponding authors.
